# Micropropagation and e*x vitro* acclimatization of *Lonicera caerulea var. altaica*: molecular identification and protocol optimization

**DOI:** 10.1038/s41598-026-43068-9

**Published:** 2026-03-05

**Authors:** Zhanargul Zhanybekova, Saltanat Bayanbay, Alevtina Danilova, Akzhunis Imanbayeva, Аlmagul Kakimzhanova

**Affiliations:** 1 National Center for Biotechology, Astana, Kazakhstan; 2 RSE “Altai Botanical Garden”, Ridder, East Kazakhstan region Kazakhstan; 3 RSE “Mangyshlak Experimental Botanical Garden”, Aktau, Kazakhstan; 4 LLP «Greenlab», Astana, Kazakhstan

**Keywords:** *L. caerulea var. altaica*, Micropropagation, DNA barcoding, *rbc*l, matK, Conservation, Biotechnology, Plant sciences

## Abstract

*L. caerulea var*. *altaica* occurs within diverse ecosystems of the Altai region and is highly valued for its frost tolerance and beneficial bioactive compounds. This study developed an efficient protocol for micropropagation and *ex vitro* acclimatization of valuable honeysuckle species. Species identification was performed using a dual DNA barcode approach with the combined *rbcL* and *matK* markers. Phylogenetic analysis revealed that specimen ABG_LA_kz grouped with the epitype, verifying its taxonomic identity. Shoot multiplication was conducted on QL medium supplemented with 0.5 mg·L⁻¹ 6-BAP, 0.2 mg·L⁻¹ GA₃, and 0.01 mg·L⁻¹ IBA. After 35 days, this treatment yielded an average of 6.53 shoots per explant, with shoot height of 2.96 cm and 37 leaves per explant. For in vitro rooting, ½ QL medium comprising 1.5 mg l^− 1^ IBA proved to be most effective, with an average 4.52 roots formed per explant and rooting percentage of 83.3% within the same culture period. An *ex vitro* acclimatization protocol using a peat: perlite (3:1) resulted in an average of 12.16 roots per plantlet, with an 100% survival. Thirty five days after acclimatization, 303 seedlings were transplanted to the nurseries of the RSE on REM “Altai Botanical Garden” and RSE on REM “Mangyshlak Experimental Botanical Garden”.

## Introduction

Plants being an integral part of medicinal practices since the dawn of human civilization, serving as the foundational sources of health restoration across diverse cultures^1^. The cultivation of honeysuckle was firstly documented in 1894^2^. The genus *Lonicera* comprises approximately 200 species worldwide^3^. More than 500 accessions have been collected and preserved at the Vavilov Research Institute of Plant Industry, reflecting sustained scientific interest in this diverse group of plants^2^. In Kazakhstan, 21 *Lonicera* species have been recorded. These species occur within mountainous regions, ranging from the Altai to the Karatau mountains and the Western Tien Shan (https://tabigat.media/wildlife/opasnie-yagodi). Although most of them are ornamental, only three species are edible: *Lonicera caerulea* var. *altaica*, *L. iliensis* Pojark., and *L. pallasii Ledeb.*^4^.

Honeysuckle is valued for its frost resistance^5^, and beneficial bioactive compounds^2,5–7^. Its different parts contain varying levels of polyphenols, such as flavonoids and hydroxycinnamic acids^8^. Population-level variation in fruit yield and morphology is attributed to both environmental and genetic factors, while the age of population representatives is estimated to the range between 40 and 50 years^9^.

Although seed propagation enhances dispersal in *Lonicera* species, it is often associated with low germination rates, uneven growth, prolonged development^10^. These limitations restrict the production of uniform and high-quality planting material, highlighting the need for alternative propagation strategies.

 In vitro techniques, such as tissue culture, offer a promising approach to generate genetically uniform and disease-free plants^11–14^ and represent one of the most effective *ex situ* preservation methods, particularly for vegetatively propagated species.

Over the past decade, substantial progress has been achieved in the micropropagation of *L. caerulea L.*^15–20^, *L. caerulea var. kamtschatica*^21,22^*L. kamtschatica “Jugana”*^23^, *L. caerulea var. kamtschatica Pojark*^24^, *L. caerulea var. kamtschatica Sevast*.

^25^, *L. caerulea var. Emphyllocalyx L.*^26^, *L. edulis*^27^, *L. caerulea subsp. edulis (Turcz. ex Herder) Turcz. ex Hulten)*^28^, *L. japonica*,* L. maackii*^29^, *L. tatarica*^30^.

However, no studies worldwide have focused on the development of biotechnological approaches for the conservation and restoration of the wild medicinal species *L. caerulea var. altaica*. In this study, the application of in vitro and *ex vitro* methods offer a promising strategy for preservation and regeneration of natural populations of *L. caerulea var. altaica*.

The objective of the study was to develop a micropropagation protocol for the valuable wild species *Lonicera caerulea var. altaica* to produce healthy plants, promoting the effective conservation and reproduction of natural populations.

## Materials and methods

### Sample collection

One-year-old shoots of *L. caerulea* var. *altaica*, approximately 15–20 cm in length, were collected. Authorization for the collection was obtained in accordance with the Charter of the Altai Botanical Garden and with the approval of the Municipal State Institution “Ridder Forestry Enterprise” under the Department of Natural Resources and Environmental Management of the East Kazakhstan Region. Samples were collected in three biological replicates at the foothills of the Ivanovsky Ridge (Ridder region) on 23 May 2023 (GPS coordinates: N50°21′27″, E83°53′48″; elevation 1200 m).

**Fig. 1:**
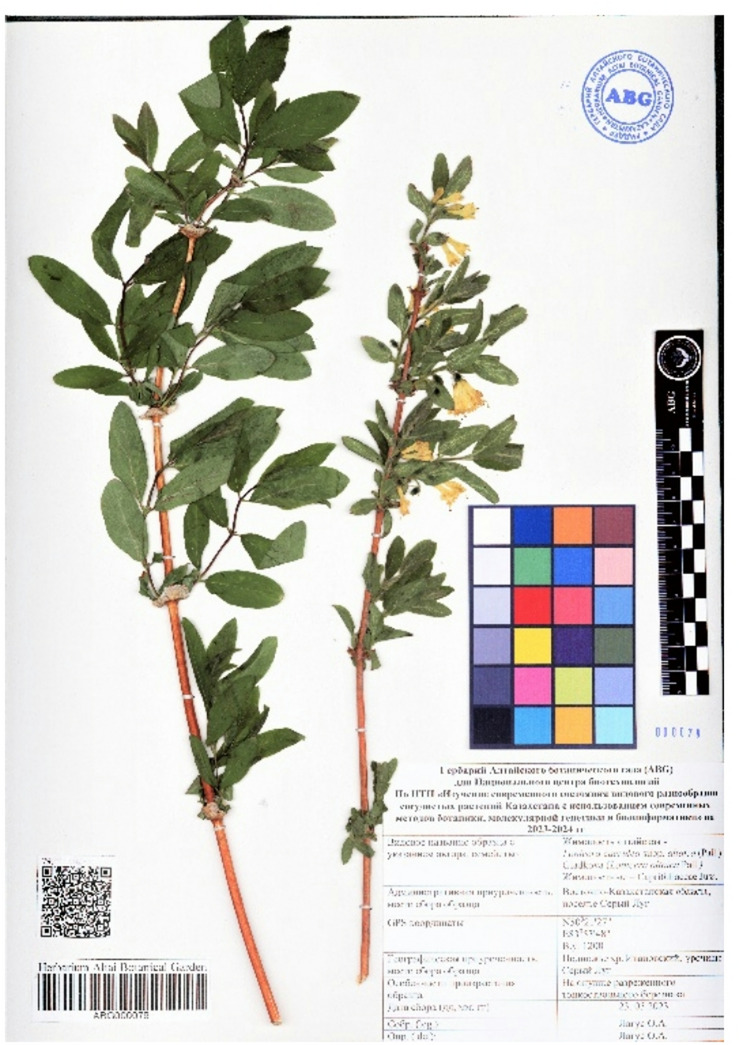
Herbarium specimen of the donor plant *L. caerulea* var. *altaica* (*Caprifoliaceae*).

The specimen was taxonomically identified by Olga Lagus, Research Scientist in the Fruit and Berry Crops Department of the Altai Botanical Garden. A voucher specimen (acronym ABG 000079) has been deposited in the public herbarium collection of the Altai Botanical Garden and is accessible through the GBIF portal (https://www.gbif.org/dataset/92a883fa-a20c-41d6-b226-0c9ab6f5b69b) (Fig. 1).

### DNA extraction, amplification and sequencing

Genomic DNA was extracted from leaves in three biological replicates using a modified cetyltrimethylammonium bromide (CTAB) method (2% CTAB, 1.4 M NaCl, 20 mM EDTA (pH 8.0), 100 mM Tris-HCl (pH 9.0)). For a pre-homogenization step, the tissue was ground using homogenizer, TissueLyser LT (Qiagen, Hilden, Germany). After grinding, the samples were incubated in CTAB extraction buffer at 65 °C for 60 min. Following this, an equal volume of chloroform was added to the sample, and the mixture was centrifuged at 20,000 rpm for 15 min at 4 °C. DNA precipitation was done by adding double cold 2-propanol volume, followed by centrifugation at 14,000 rpm for 30 min at 4 °C. The precipitate was washed twice with 70% ethanol and dried. The DNA pellet was dissolved in 30 µL 1×TE buffer (1 mM EDTA, 10 mM Tris-HCl, pH 8.0). The quality and quantity of extracted DNA were assessed using a Qubit dsDNA High Sensitivity (HS) Assay Kit and Qubit Fluorometer (Thermo Fisher Scientific, Waltham, MA, USA).

Polymerase chain reaction (PCR) amplification of the *ribulose-1*,*5-bisphosphate carboxylase/oxygenase large subunit (rbcL)* and *maturase K (matK)* genes was performed using high-quality DNA and gene-specific primers synthesized at the National Center for Biotechnology (NCB), Astana, Kazakhstan.

PCR reactions were set up in a final volume of 20 µL, containing 9.4 µL of deionized distilled water (ddH₂O), 8.6 µL of premix (0.2 µL Taq polymerase, 200 µM of each dNTP, 3.0 µL PCR buffer (10×), and 2 mM MgCl₂, 0.8 µL each of primers), and 2 µL of DNA (100 ng). Negative controls were included in each reaction set.

The PCR cycling conditions were as follows: at 94 °C for 5 min; 30 cycles of 94 °C for 30 s, 52–62 °C (depending on the primers) for 40 s, 72 °C for 50 s; and 72 °C for 7 min. PCR products were resolved on a 1.5% agarose gel stained with ethidium bromide (EtBr) for 40 min. 1 Kb DNA ladder (Thermo Fisher Scientific, USA) was used as a molecular weight marker. The results were documented using a gel documentation system (Bio-Rad Laboratories, Hercules, CA).

DNA fragments were sequenced using the Sanger sequencing method, following the manufacturer’s instructions. Subsequently, sequence similarity searches were performed using the Nucleotide BLAST program at the National Center for Biotechnology Information (NCBI) website (http://blast.ncbi.nlm.nih.gov/Blast.cgi) to identify homologous sequences.

### Phylogenetic analysis

The nucleotide sequences of the *rbcL* and *matK* regions were aligned using the MUSCLE algorithm. Phylogenetic analysis was conducted using the maximum likelihood (ML) method with the Kimura 2-parameter model, and the robustness of the tree was assessed with 1,000 bootstrap replicates in MEGA11 (version 11.0.13). Sites containing gaps or missing data were excluded using the complete deletion option.

### Culture establishment

Axillary buds were stored at 4 °C until use. Subsequently, they were excised from nodal segments, with each segment measuring 2–3 cm in length. The nodal segments were subjected to sterilization treatments using soapy water three times, followed by washing under the running tap water for 30 min.

Further, 5%, 10% and 15% H_2_O_2_ (Labkhimprom LLP, Almaty, Kazakhstan) solutions for 5 min were examined under sterile conditions in laminar flow (ESCO Global, Changi South Street 1, Singapore). The explants were shaken continuously during the sterilization and rinsed with sterile distilled water three times.

For shoot initiation, axillary buds were cultured on Quoirin and Lepoivre (QL)^31^, Murashige and Skoog (MS)^32^, and Woody Plant Medium (WPM)^33^ media without plant growth regulators (PGRs) to select the optimal medium for multiplication and rooting.

The number of buds obtained during the sterilization and regeneration stages was recorded to calculate the percentage of viable explants after 28 days.

### Shoot multiplication

Regenerated shoots ranging from 1.0 to 2.28 cm in length were cultured on QL medium supplemented with 30 g L ⁻¹ sucrose and solidified with 7 g L⁻¹ agar. This medium was further supplemented with various concentrations of plant growth regulators (PGRs, Sigma-Aldrich, Shanghai, China) such as 6-benzylaminopurine (6-BAP) at four different levels, in combination with constant concentrations of indole-3-butyric acid (IBA) and gibberellic acid (GA_3_) to facilitate effective shoot multiplication (Table 1).

**Table 1:** Combinations of growth regulators for multiplication.

Treatment	6-BAP (mg L⁻¹)	GA_3_ (mg L⁻¹)	IBA(mg L⁻¹)
I	–	–	–
II	0.2	0.2	0.01
III	0.5		
IV	0.75		

The pH of the medium was carefully adjusted to a range of 5.80 using 1 M HCl/NaOH prior to autoclaving at 121 °C for 20 min (MELAtronic 23, Berlin, Germany), before adding 7 g L⁻¹ agar (Sigma-Aldrich Chemie GmbH, Taufkirchen, Germany). In our study, 30 explants were cultivated for each treatment, and data was collected after 35 days cultivation. The cultures were incubated in a climate chamber at 24–26 °C, with a relative humidity of 60–80%. A photoperiod of 16 h light/8 h dark was maintained. Shoot multiplication was carried out through four in vitro subcultures.

### Rooting of in vitro shoots and acclimatization

Shoots, approximately 2.0–3.6 cm in length, were cut off the explants and transferred to half-strength QL medium (½ QL) supplemented with 0.5, 1.0, or 1.5 mg L⁻¹ IBA. PGR-free medium served as the control. For each treatment, 30 explants were cultivated. The cultures were maintained in a climate chamber at 24–26 °C with 60–80% relative humidity under a 16 h light/8 h dark photoperiod. Data was recorded after 35 days cultivation.

The rooted plantlets were washed thoroughly to remove any medium residues and potted into the mixture from different substrates, such as, neutral peat (high peat (90%) and low peat (10%), pH-4.5-5.5, Kekkilä Oy, Vantaa, Finland), high peat (high peat (100%), pH-5.9, Kekkilä Oy, Vantaa, Finland), vermiculite (Sadovita LLC, Penza, Russia), and perlite (Agroperlite, Borresources LLC, Bor, Russia). The following treatments were studied, as presented in Table 2.

**Table 2:** Composition of substrate mixtures used for plantlet transplantation.

Treatment	Substrate mixture
I	Neutralized peat
II	Peat: perlite 3:1 (v/v)
III	Peat: vermiculite 3:1 (v/v)
IV	High peat

Each treatment consisted of 25 plants. To maintain relative humidity at approximately 80–90% and temperatures between 25 and 28 °C, the plants were covered with transparent plastic containers, which were removed after 14 days to allow gradual acclimatization. Plants were watered with 100 mL of tap water per three days. Growth parameters, including primary root length, the number of adventitious roots, a shoot length, and the number of shoots, were recorded 35 days after transplantation.

### Experimental design and statistical analysis

The experiment was conducted using a completely randomized block design with three replications. Assumptions of normality and homogeneity of variances were assessed using the Shapiro-Wilk and Brown-Forsythe tests, respectively. For normally distributed data with unequal variances, Welch’s ANOVA was applied, while the Kruskal-Wallis test was used for non-parametric data. Significant differences were identified using post-hoc tests. All statistical analyses were two-tailed and performed at a significance level of α = 0.05 (95% confidence interval). Data are presented as means ± standard error (SE) from three independent experiments.

## Results

### DNA barcoding of *L. caerulea var. altaica and* phylogenetic analysis

The extracted Genomic DNA exhibited sufficient quality for downstream applications and was successfully amplified using universal chloroplast markers targeting the *rbcL* and *matK* regions. The resulting amplicons were 464 bp for *rbcL* and 849 bp for *matK*. The sequences analyzed in the current study are available in the GenBank database under the accession numbers *rbcL* – PV642429, *matK* – PX439459.

To ensure taxonomic identification, a multilocus phylogenetic analysis based on the combined *rbcL* and *matK* data was performed using reference sequences of *Lonicera*-related taxa retrieved from GenBank (Table 3).

**Table 3:** Accessions of *Lonicera* taxa retrieved from GenBank.

Voucher/ Isolate	Family/taxon	NCBI, Accession number rbcL	NCBI, Accession number matK
ABG_LA_kz	*L. caerulea var. altaica*	PV642429	PX439459
18.2.1.1	*L. caerulea var. altaica*	PV026015	PV026015
Q799	*L. ligustrina*	MH658714	MH660179
YLDP139C	*L. lanceolat*	MH116245	MH116704
Q189	*L. tragophylla*	MH658149	MH659632
Ge130823b	*L. tragophylla*	MH657429	MH659000
P._L._Liu_264	*L. tragophylla*	MN722338	MN722232
YLDP108A	*L. angustifolia var. myrtillu*	MH116242	MH116702
PS1160MT01	*L. hypoglauca*	HM228474	HM228430
Q440	*L. tangutica*	MH658381	MH659859
YLDP001C	*L. tangutica*	MH116251	MH116710
Q313	*L. tangutica*	MH714061	MH714248
Q070	*L. maackii*	MH658036	MH659521
P._L._Liu_231	*L. fragrantissima*	MN722341	MN722234
MO: Carlsen3280	*L. acuminata*	OL536976	OL690053
Q823	*L. acuminata*	MH658735	MH660199
10cs2264	*L. trichosantha*	MN185140	MN267141
T._T._Tian_3	*L. ferdinandi*	MN722339	MN722237
TLF-178	*L. chrysantha*	MT931254	MT918125
Ge130661	*L. hispida*	MT931257	MT918124.1
Q189	*L. tragophylla*	MH658149	MH659632
Ge130823b	*L. tragophylla*	MH657429	MH659000
Liu 264	*L. tragophylla*	MN722338	MN722232
JAG 0228	*Diervilla lonicera*	MH657429	MH657429
BOP012296	*Weigela florida*	KP297684	KP297538

**Fig. 2:**
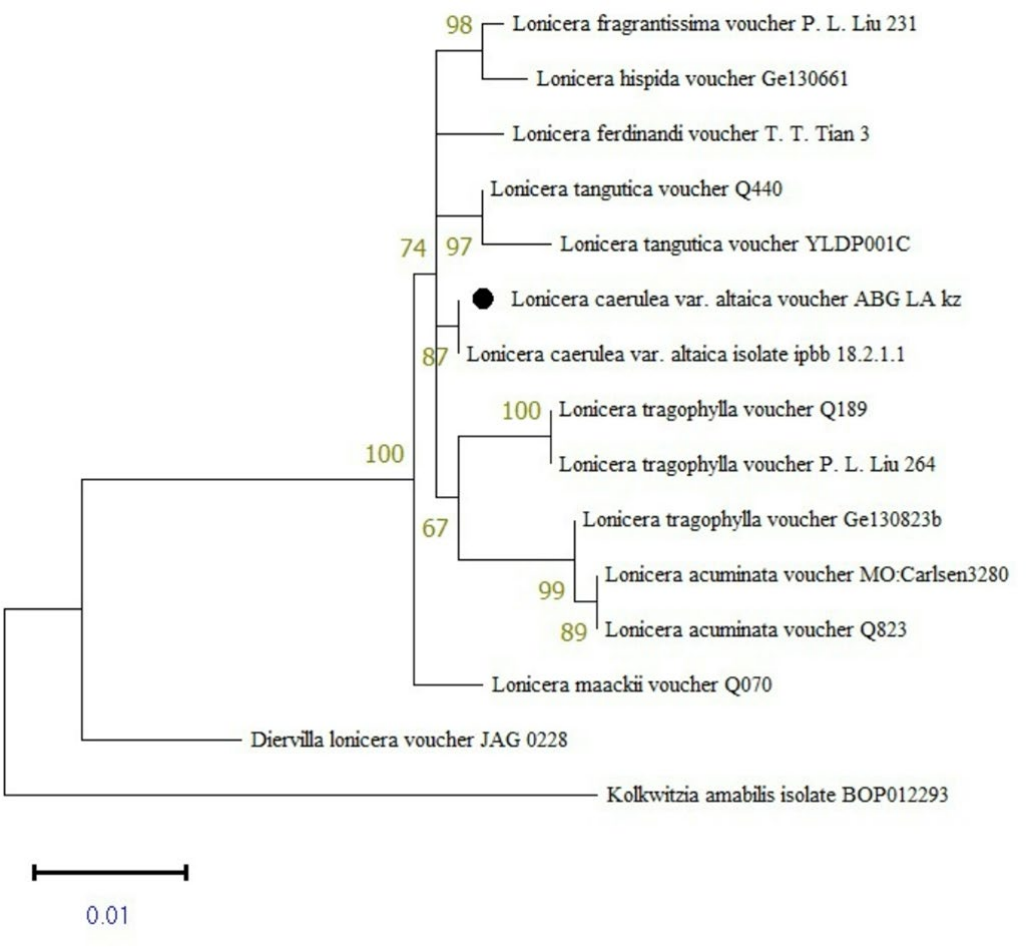
Phylogenetic tree reconstructed by the ML method based on combined barcode data (*rbcL*+*matk*) for seven *Lonicera* species using MEGA11 (version 11.0.13). with 1000 bootstrap replications. Bootstrap values ≥ 60% were considered as significant and are indicated in the phylogenetic tree.

The inferred phylogenetic topology resolved several well-supported groups corresponding to different *Lonicera* species, as indicated by high bootstrap values. The specimen ABG_LA_kz, identified as *L. caerulea var*. *altaica*, combined with the reference isolate (IPBB 18.2.1.1) confirming its taxonomic identity (Fig. 2).

### Establishment of *L. caerulea subsp. altaica* in vitro culture

**Fig. 3:**
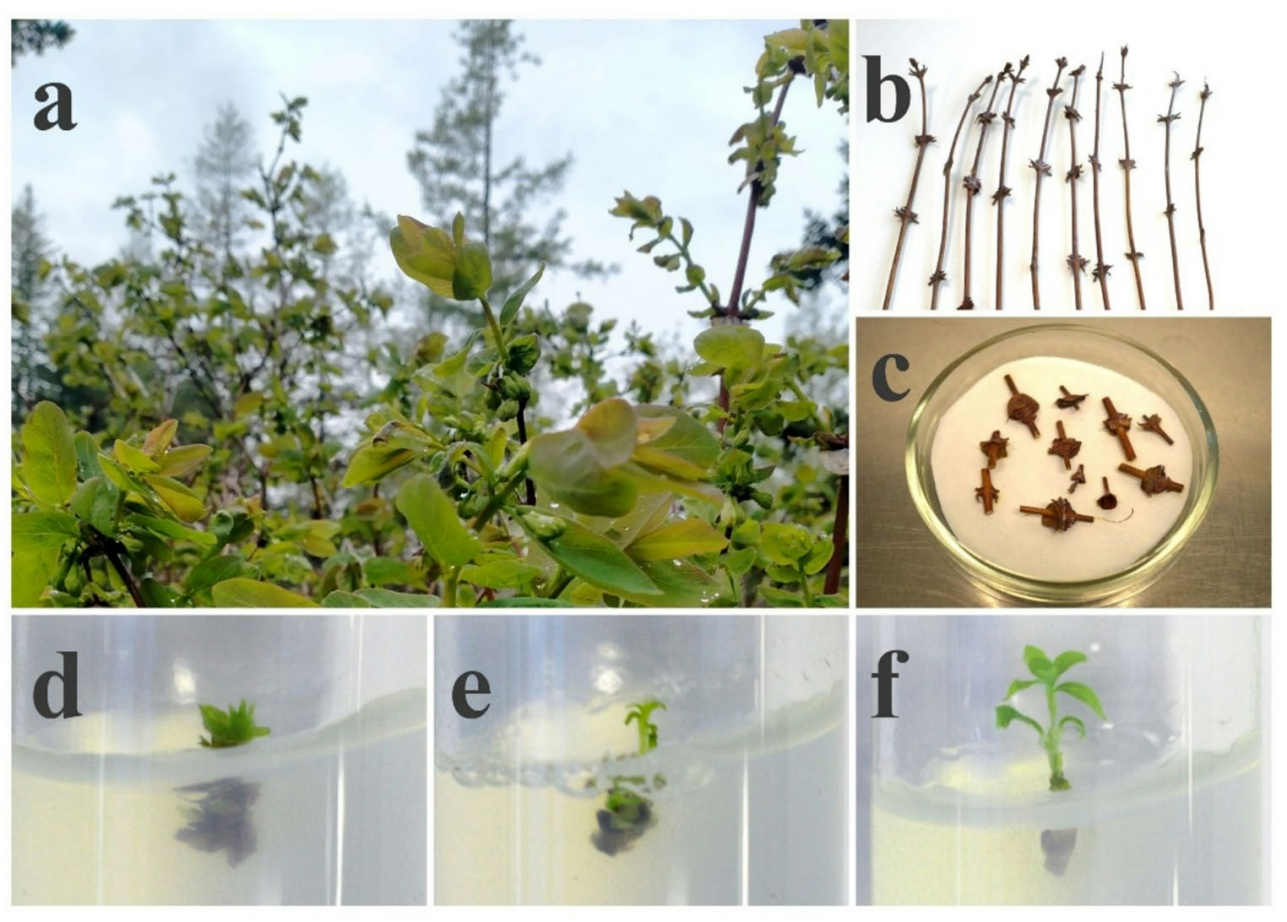
Establishment of in vitro culture. (**a**) Donor plant of *L. caerulea* var. *altaica*; (**b**) One-year-old shoots; (**c**) Excised shoot segments; (**d)** Bud induction in MS; (**e**) Bud induction in WPM; (**f)** Bud induction in QL.

For the establishment of in vitro cultures, one-year-old shoots, approximately 15–20 cm in length, taken from a donor plant were divided into single-node segments (2–3 cm), each containing one axillary bud (Fig. 3a–c). After the surface sterilization, the explants were cultured on PGR-free medium.

The results indicate the treatment with 15% H₂O₂, as a sterilizing agent, provides a high level of explant sterility and supports successful regeneration, and, thus, can be recommended for establishing aseptic cultures (Table 4).

**Table 4:** Results of axillary bud sterilization.

Treatment	Explants, pcs	Contamination, %	Necrosis, %	Viability, %
5% H_2_O_2_	30	100	**–**	–
10% H_2_O_2_	30	66.7	–	33.3
15% H_2_O_2_	30	10	10	80.0

Following the optimized sterilization process, three PGR-free nutrient media were evaluated to promote effective shoot regeneration. Among these, QL medium demonstrated superior performance, yielding significantly at higher rates after 28 days of cultivation. Specifically, the QL medium achieved the regeneration rate of 60% (18 out of 30 explants), when MS medium showed a response rate of only 30% (9 out of 30), and WPM medium resulted in a 46.6% response rate (14 out of 30). While explants maintained on MS and WPM media exhibited reduced vigor and poor vitality (Fig. 3d, e), those cultured on QL medium demonstrated superior growth, appearing healthier and more vigorous (Fig. 3f). Accordingly, QL medium was selected as the most suitable one for subsequent micropropagation experiments.

### Optimization of PGRs for in vitro propagation of *L. caerulea* var. *altaica*

**Fig. 4:**
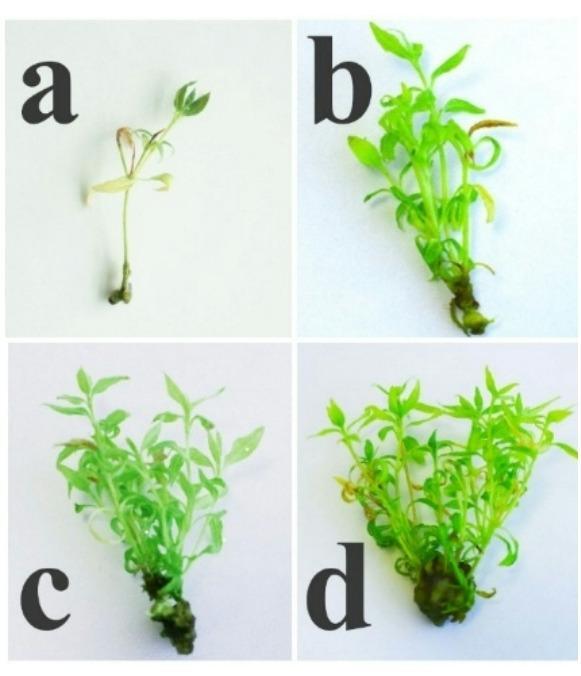
Effect of 6-BAP concentrations on shoot multiplication of *L. caerulea* var. *altaica* after 35 days (**a**) PGRs free; (**b**) 0.2 mg L⁻¹ 6-BAP, 0.2 mg L⁻¹ GA₃ and 0.01 mg L⁻¹ IBA; (**c**) 0.5 mg L⁻¹ 6-BAP, 0.2 mg L⁻¹ GA₃ and 0.01 mg L⁻¹ IBA; (**d**) 0.75 mg L⁻¹ 6-BAP, 0.2 mg L⁻¹ GA₃ and 0.01 mg L⁻¹ IBA.

To assess microshoot propagation, the effects of different concentrations of 6-BAP with GA₃ and IBA were evaluated using QL medium. The results demonstrated that optimization of exogenous PGRs is crucial to be a success in vitro culture (Table 5; Fig. 4a–d). Studies at the shoot multiplication stage yielded statistically significant improvements, leading to an increased multiplication coefficient.

In our study, Treatment IV resulted in the highest shoot multiplication, with an average of 8.32 ± 0.18 shoots per explant, 77.57 ± 2.25 leaves per plant, and an average shoot height of 2.53 cm. However, 6-BAP concentration of 0.75 mg L⁻¹ induced increased callus formation.

Treatment III produced microshoots of satisfactory morphological quality, with 6.53 shoots per explant (an increase of 5.53), showing significantly higher performance compared to the control and other treatments. Notably, the highest average shoot length of 3.73 cm was obtained with a combination of 0.2 mg L⁻¹ 6-BAP, 0.2 mg L⁻¹ GA₃, 0.01 mg L⁻¹ IBA.

**Table 5:** Effect of PGRs concentrations on shoot multiplication of *L. caerulea* var. *altaica.*

Treatment	PGR (mg L⁻¹)			35 days after cultivation			Increase		
	6-BAP	GA₃	IBA	Shoot height, (cm)	Number of shoots per explant	Number of leaves per explant	Shoot height, (cm)	Number of shoots per explant	Number of leaves per explant
I	–	–	–	2.53 ± 0.14	1.23 ± 0.07	7.43 ± 0.17	1.06	0.23	1.49
II	0.2	0.2	0.01	3.73 ± 0.13****	3.93 ± 0.17****	27.57 ± 1.86****	2.22	2.93	21.87
III	0.5			2.96 ± 0.06*	6.53 ± 0.27****	37.00 ± 1.19****	1.47	5.53	30.96
IV	0.75			2.53 ± 0.10	8.32 ± 0.18****	77.57 ± 2.25****	0.99	7.32	71.90

Increase refers to the absolute difference between the final and baseline values.

*, ****p < 0.05, 0.0001, respectively.

QL medium (Treatment III) supplemented with 0.5 mg L⁻¹ 6-BAP, 0.2 mg L⁻¹ GA₃, and 0.01 mg L⁻¹ IBA was identified as the optimal combination, resulting in significant increases in shoot height, number of shoots, and number of leaves, while maintaining satisfactory microshoot quality for rooting./ v.

### *In* vitro rooting and acclimatization of *L. caerulea var. altaica*

Rooting is a critical stage in the clonal micropropagation of plants, serving as a key determinant for the successful acclimatization of in vitro cultured plantlets to ex vitro conditions. The capacity of plantlets to form roots reflects their readiness to be transferred to soil that directly affects their growth, adaptability, and subsequent performance.

**Fig. 5:**
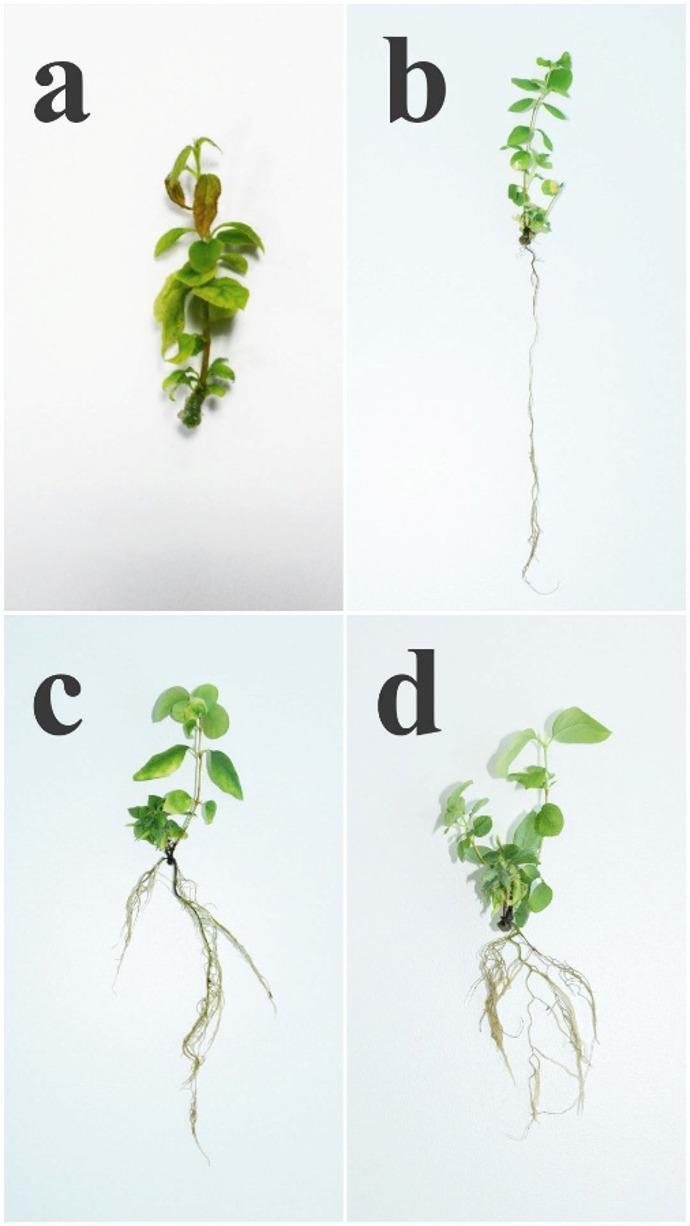
Effect of different IBA concentrations on in vitro rooting of *L. caerulea var. altaica* after 35 days (**a**) PGR-free medium; (**b**) 0.5 mg L⁻¹ IBA; (**c**) 1.0 mg L⁻¹ IBA; (**d**) 1.5 mg L⁻¹ IBA.

In this study, shoots of L. caerulea var. altaica were cultured on basal ½ QL medium supplemented with varying IBA levels (Table 6; Fig. 5a–d). A low concentration of 0.5 mg L⁻¹ IBA resulted in a reduced rooting response (53.3%) and fewer roots per shoot (1.18 ± 0.10), the roots were thinner and significantly longer (8.93 ± 0.27 cm). In contrast, shoots treated with 1.5 mg L⁻¹ IBA developed 4.52 ± 0.21 roots per shoot, with 83.3% rooting success and achieving a mean root length of 7.34 ± 0.35 cm within 35 days. The shoots increased in height by 4.05 cm and produced an average of 6.61 new leaves per shoot.

Thus, to obtain in vitro plantlets with a well-developed root system, the most suitable medium is ½ QL supplemented with 1.5 mg L⁻¹ IBA.

**Table 6:** Effect of different IBA concentrations on root development of *L. caerulea var. altaica.*

Treat ment	IBA (mg L⁻¹)	35 days after cultivation					Increase	
		Shoot height, (cm)	Number of leaves	Number of roots	Root length, (cm)	Rooting percentage, %	Shoot height, (cm)	Number of leaves
I	–	5.42 ± 0.10	8.7 ± 0.24	0.16 ± 0.06	0.03 ± 0.01****	–	2.49	2.03
II	0.5	6.05 ± 0.27	11.22 ± 0.34****	1.18 ± 0.10*	8.93 ± 0.27****	53.3	3.05	4.95
III	1.0	6.54 ± 0.23***	12.00 ± 0.52****	2.57 ± 0.22****	7.70 ± 0.32****	70.0	3.58	5.66
IV	1.5	6.93 ± 0.30****	13.41 ± 0.71****	4.52 ± 0.21****	7.34 ± 0.35****	83.3	4.05	6.61

Increase refers to the absolute difference between the final and baseline values.

*, ***, ****p < 0.05, 0.001, 0.0001, respectively.

The successful hardening and acclimatization of plantlets obtained through in vitro culture in their natural habitat are a critical step in the propagation and conservation of *L. caerulea* var. *altaica*. Furthermore, factors such as medium pH and substrate composition play a key role in facilitating the adaptation and survival of micropropagated plantlets.

**Fig. 6:**
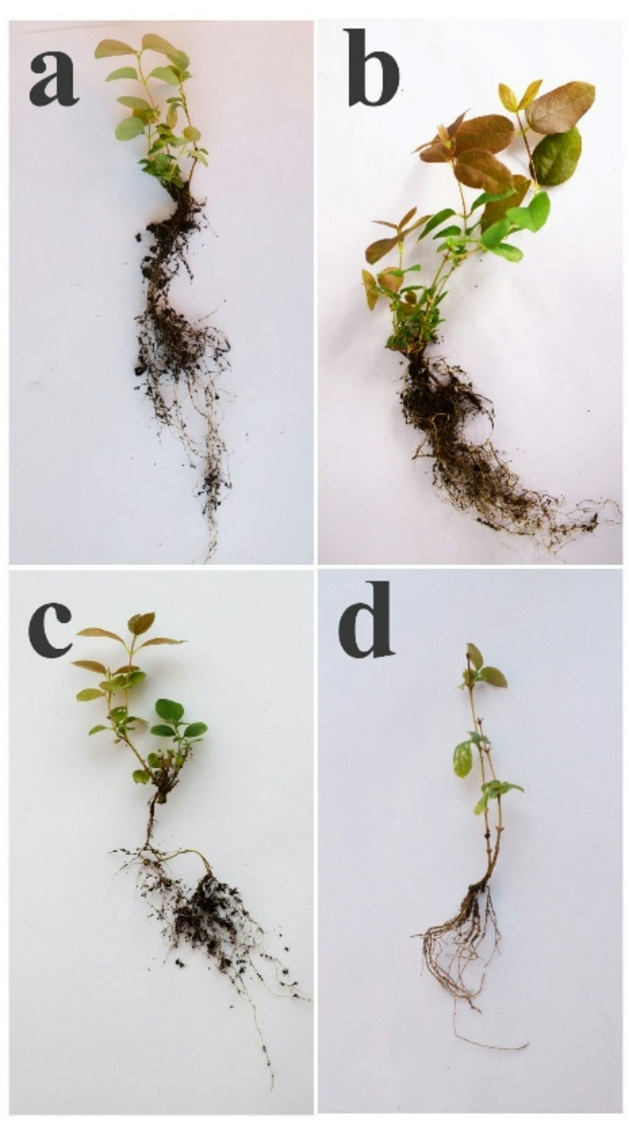
Acclimatization of *L. caerulea* var. *altaica* plantlets (**a**) neutral peat; (**b**) peat: perlite (3:1 *v/v*); (**с**) peat: vermiculite (3:1 *v/v*); (**d**) high peat.

To further acclimatize the rooted shoots, four different substrates were evaluated with the result ranging from 72% to 100% survival rate (Table 7; Fig. 6a–d). Among these substrates, the combination of peat and perlite (3:1 v/v) proved to be the most effective, yielding an average plant height of 10.52 ± 0.27 cm, 31.56 ± 1.96 leaves per shoot, 12.16 ± 0.44 roots per shoot, and 100% survival rate. Neutral and lowland peat compositions enhance the substrate’s water retention and buffering capacity. Additionally, the fine particle size fraction up to 6 mm improved water absorption and promoted better root system development.

The least response was recorded for the treatment with top peat where only 1.61 ± 0.14 shoots per explants and 9.61 ± 1.00 leaves per shoot with decreased root length 7.16 ± 0.68 cm with 72% survival rate. This can be explained by the coarse particle size fraction of up to 15 mm, which absorbs and retains water less effectively, resulting in weaker plants.

Thus, we observed that neutral peat combined with perlite was optimal for acclimatization of *L. caerulea* var. *altaica.*

**Table 7:** Effect of substrates on acclimatization of *L. caerulea* var. *altaica* plantlets 35 days after cultivation.

Substrate	Shoot height, (cm)	Number of shoots	Number of leaves	Root length, (cm)	Root number	Survival rate, %
Neutral peat	8.08 ± 0.34	3.04 ± 0.17	25.04 ± 1.16	14.32 ± 0.20	6.08 ± 0.24	96
Peat: perlite (3:1 )	10.52 ± 0.27***	4.04 ± 0.16**	31.56 ± 1.96*	13.48 ± 0.55	12.16 ± 0.44****	100
Peat: vermiculite (3:1 *v/v*)	9.10 ± 0.39	3.36 ± 0.19	26.56 ± 1.34	11.69 ± 0.25***	5.32 ± 0.23	100
Top peat	6.55 ± 0.33	1.61 ± 0.14***	9.61 ± 1 0.00****	7.16 ± 0.68****	6.11 ± 0.32	72

*, **, ***, **** *p* < 0.05, 0.01, 0.001, 0.0001, respectively.

**Fig. 7:**
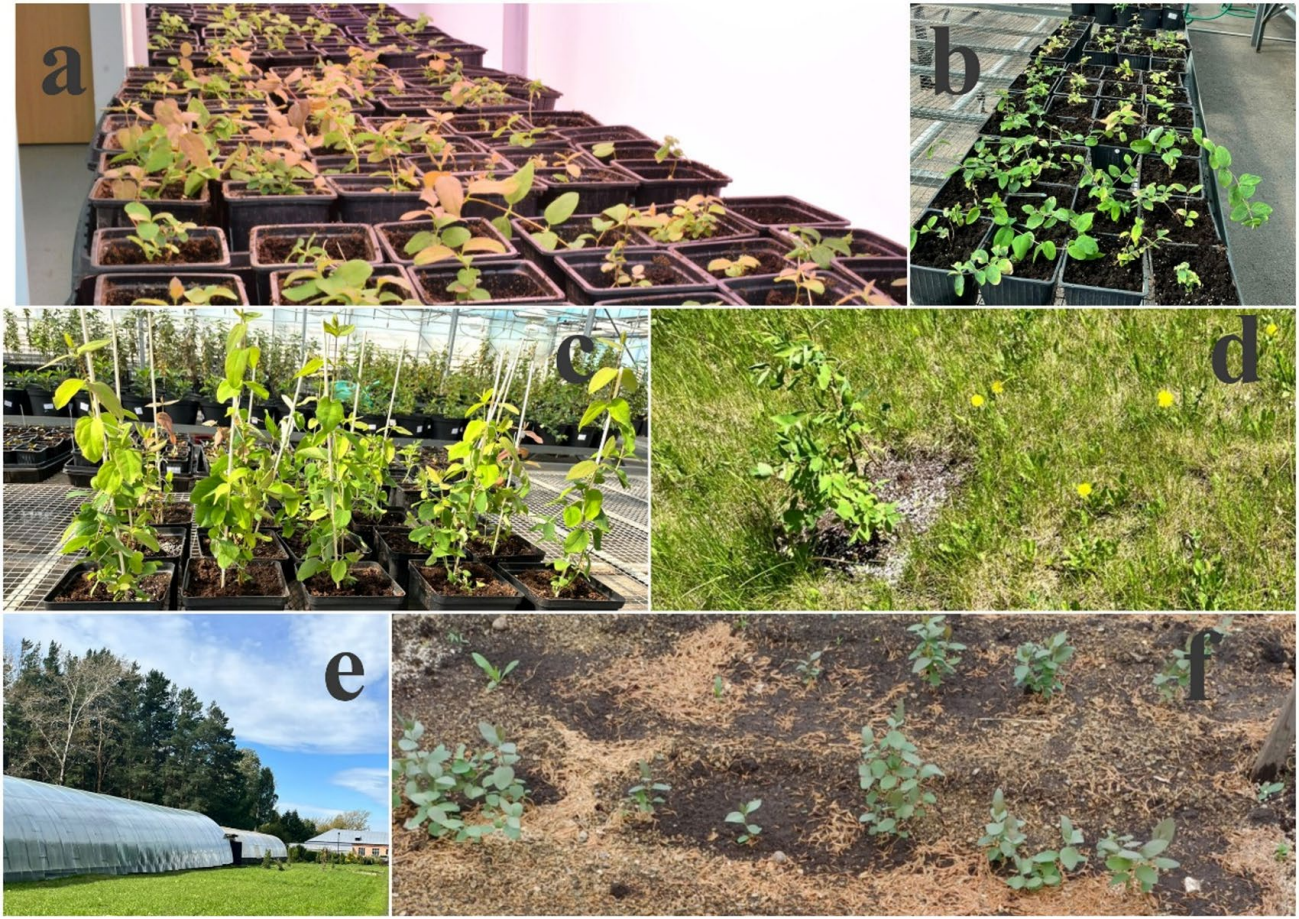
Acclimatization of *L. caerulea* var. *altaica* plantlets (**a**) growth room conditions; (**b**, **c**) greenhouse conditions (NCB, Astana); (**d**) transferred plantlets in the open ground (NCB, Astana); (**e**,** f**) transferred plantlets in the natural substrate in RSE on REM “Altai Botanical Garden”.

A total of 303 miniseedlings of *L. caerulea* var. *altaica* were transferred from the growth room (Fig. 7a) to the greenhouse after 35 days of acclimatization (Fig. 7b, c). Subsequently, the plantlets were transplanted into the open ground at the NCB (Fig. 7d) and at the Republican State Enterprise on the Right of Economic Management (RSE on REM) “Altai Botanical Garden” (Fig. 7e, f), as well as at the RSE on REM “Mangyshlak Experimental Botanical Garden”.

## Discussion

To conserve and restore natural populations of medicinally valuable wild shrub species such as *L. caerulea* var. *altaica*, a micropropagation protocol based on axillary buds was developed. This protocol comprised four main stages: culture establishment, shoot proliferation, rooting, and acclimatization. Various combinations of exogenous PGRs and substrates were evaluated to produce healthy, acclimatized plantlets genetically identical to the donor plant. The successfully propagated plants were subsequently introduced to the RSE on REM “Altai Botanical Garden” and the “Mangyshlak Experimental Botanical Garden” for further growth and conservation.

### Molecular identification

The species identity of the donor plant was confirmed using the molecular markers *rbcL* and *matK*. The resulting DNA sequences were deposited in the NCBI database and assigned accession numbers. In plant DNA barcoding, multiple genomic regions are employed to improve species discrimination, with commonly used markers including plastid sequences such as, *trnL*^34^, *rbcL*,* matK* and *trnH-psbA*, as well as the nuclear ribosomal *ITS* region^35^.

By 2009, *rbcL* and *matK* had become the widely accepted dual-locus barcodes for global plant databases^36^, and provide an acceptable rate of species resolution in flowering plants^37,38^.

In our study, multilocus analysis using the *matK* and *rbcL* regions with *Lonicera*-related references showed that specimen ABG_LA_kz grouped with reference isolate IPBB 18.2.1.1, confirming its taxanomic identity.

### Establishment of in vitro culture

Numerous studies have reported micropropagation protocols for various *Lonicera species*^15–17,19,20,26^.

However, published data on *L. caerulea subsp. altaica* remains limited. Consequently, our findings are compared with published data from closely related species.

For in vitro culture initiation, the use of 5–24% H₂O₂ solution during explant sterilization resulted in a high yield of viable explants, reaching 73.3% for certain medicinally, economically important, rare, endangered plant species^39–42^.

Among the tested concentrations, 15% H₂O₂ solution proved most effective, with 80% yield of sterile explants. After the sterilization stage, the choice of basal medium becomes a critical extrinsic factor influencing subsequent explant development. Differences in explant growth responses have been attributed primarily to variations in macroelement composition^43^. In this study, we used QL medium with potassium nitrate (1800 mg L⁻¹), absent in WPM and calcium nitrate (578.92 mg L⁻¹). Media with appropriate nitrate sources can enhance shoot regeneration^43^.

### Shoot multiplication

Successful explant development is determined by both intrinsic and extrinsic regulatory factors^44–46^.

PGRs play a crucial role in regulating shoot multiplication by modulating endogenous hormone homeostasis, influencing the expression of hormone-related genes^47^, and interacting with photoreceptor-mediated signaling pathways to regulate proliferation^48^. For instance, 6-BAP has emerged as a widely recognized and effective PGR^49–52^, which activates cytokinin signaling pathways that facilitate direct transcriptional activation of WUSCHEL (*WUS)* through type-B *ARR* binding and favorable epigenetic modifications, thereby initiating axillary meristem development^53^.

Nevertheless, the effectiveness of 6-BAP varies considerably across genotypes. Higher 6-BAP concentrations (2.0 mg L⁻¹) were applied to achieve moderate proliferation in Clones 44 and 46, whereas other genotypes responded at lower concentrations^17^.

Beyond adenine-derived cytokinin, thidiazuron (TDZ) provides alternative options for enhancing shoot proliferation. Owing to their relative resistance to degradation by cytokinin oxidase/dehydrogenase (*CKX*), this cytokinin can exhibit prolonged bioactivity^54^.

However, despite the high efficiency of TDZ^55^, multiple reports have reflected its ability to induce vitrification^47^ and abnormalities^56^.

Several studies on *Lonicera* have emphasized that optimal shoot proliferation depends not on individual PGR alone, but on their combined application, underscoring the importance of hormone signaling cross-talk^10,15,18,27^. GA₃-mediated stem elongation occurs through interaction with the GIBBERELLIN INSENSITIVE DWARF1 (*GID1*) receptor, which relieves *DELLA* repression either by promoting proteolytic degradation of DELLA repressors or by directly inhibiting DELLA activity^57^. The co-application of GA_3_ (3.46 mg L⁻¹) and BA (2.25 mg L⁻¹) showed high effectiveness in promoting lateral bud outgrowth in *J. curcas*^58^. Supporting previous finding on the positive effect of combined PGRs application, our results demonstrated a synergistic effect of BAP (0.5 mg L⁻¹), GA₃ (0.2 mg L⁻¹), and IBA (0.01 mg L⁻¹), yielding shoots with an average height of 2.96 cm, 6.53 shoots per explant, and 37 leaves per explant.

### Rooting

 In vitro root formation is one of the essential steps, because root quality directly determines plant survival in *ex vitro* conditions. The efficacy of various auxins for in vitro rooting in *Lonicera* species has been widely investigated^10,15,19–22,30^.

In particular, IBA is consistently reported as one of the most effective auxins for root induction across diverse plant species^15,16,59^. Its supplementation in vitro provides a controlled auxin source, and its physiological effects are largely attributed to its conversion to indole-3-acetic acid (IAA)^60^, which stimulates cell division, elongation, and differentiation, leading to adventitious and lateral root formation, meristem activity, and root hair development^61^.

Previous study has shown that 1.0 mg L⁻¹ IBA effectively promotes rooting in *Lonicera* genotypes^19,22^. In our study, 1.5 mg L⁻¹ IBA resulted in an average of 4.52 roots per explant. In contrast, Kadim et al.^15^ recorded a much higher number of roots (28.30 per explant) at the same concentration, although root length (4.80 cm) was shorter compared to our results (7.34 cm).

### Acclimatization

The acclimatization stage is the most complicated phase in micropropagation, when plants are moved into *ex vitro* environment. This frequently induces stress that can reduce the viability of regenerants and sometimes lead to mortality^19^. *Lonicera* species demonstrate considerable ecological plasticity at the genus level^62^.

Previous research on *L. caerulea* cultivars demonstrated high viability and cultivar-specific growth using a mixture of peat, sand, top soil, and perlite (1:1:1:1)^19^. In our study, a substrate composed of peat and perlite (3:1) proved most effective for acclimatization of *Lonicera caerulea var. altaica*, resulting in 100% survival rate. Similarly, *L. caerulea*^21^ and *L. edulis*^62^ demonstrated a preference for peat- and perlite-based substrates.

The limitation of this study is the lack of a post-propagation genetic stability assessment. However, the use of meristem-based axillary bud culture in perennial species^63^, the absence of a callus phase^64^, and the short culture period^65^ can provide genetic fidelity. In addition, previous studies reported that micropropagated plants were genetically stable and showed high genetic homogeneity^66–68^.

## Conclusion

A protocol for micropropagation and *ex vitro* acclimatization of the valuable medicinal wild species *L. caerulea* var. *altaica*, native to eastern Kazakhstan, was developed for conservation and propagation purposes. The use of a two-barcode multilocus approach (matK+rbcL) confirmed the identity of *L. caerulea* subsp. *altaica*. In this study, after 35 days, an average of 6.53 microshoots per explant were obtained on QL medium supplemented with 0.5 mg L^− 1^ BAP, 0.2 mg L⁻¹ GA₃, and 0.01 mg L⁻¹ IBA. In vitro rooted plantlets were successfully acclimatized under *ex vitro* conditions, achieving a maximum survival rate within 35 days. This protocol can be applied for propagation of this valuable medicinal wild species to support its conservation.

## Data Availability

The sequences generated during the current study are available in the GenBank, [https://www.ncbi.nlm.nih.gov/nuccore/PV642429, https://www.ncbi.nlm.nih.gov/nuccore/PX439459.1/].
